# Energy landscape-driven non-equilibrium evolution of inherent structure in disordered material

**DOI:** 10.1038/ncomms15417

**Published:** 2017-05-19

**Authors:** Yue Fan, Takuya Iwashita, Takeshi Egami

**Affiliations:** 1 Department of Mechanical Engineering, University of Michigan, Ann Arbor, Michigan 48109, USA; 2 Division of Natural Sciences, Oita University, Oita-shi 870-1192, Japan; 3 Shull Wollan Center – Joint Institute for Neutron Sciences, Knoxville, Tennessee 37831, USA; 4 Department of Materials Science and Engineering, University of Tennessee, Knoxville, Tennessee 37996, USA; 5 Department of Physics and Astronomy, University of Tennessee, Knoxville, Tennessee 37996, USA; 6 Materials Science and Technology Division, Oak Ridge National Laboratory, Oak Ridge, Tennessee 37831, USA

## Abstract

Complex states in glasses can be neatly expressed by the potential energy landscape (PEL). However, because PEL is highly multi-dimensional it is difficult to describe how the system moves around in PEL. Here we demonstrate that it is possible to predict the evolution of macroscopic state in a metallic glass, such as ageing and rejuvenation, through a set of simple equations describing excitations in the PEL. The key to this simplification is the realization that the step of activation from the initial state to the saddle point in PEL and the following step of relaxation to the final state are essentially decoupled. The model shows that the interplay between activation and relaxation in PEL is the key driving force that simultaneously explains both the equilibrium of supercooled liquid and the thermal hysteresis observed in experiments. It further predicts anomalous peaks in truncated thermal scanning, validated by independent molecular dynamics simulation.

## Abstract (web summary)

Fundamental understanding of glass dynamics is challenging owing to their complex non-equilibrium nature and thus the multi-dimensional potential energy landscape. Here, Fan *et al*. present a model to explore the glass energy landscape driven by thermal activation and relaxation, which are temporally decoupled.

Glasses do not have structural defects found in crystalline materials, such as dislocations and grain boundaries. For this reason glasses have many promising physical, chemical and mechanical properties in comparison to their crystalline counterparts^1^,^2^,^3^, making them attractive for a wide range of applications. Many properties of glassy materials are very sensitive to the degree of relaxation in the systems, which can be expressed in terms of their inherent structure (IS) obtained by removing the kinetic energy^4^,^5^,^6^. However, a fundamental understanding is still lacking on the dynamics of IS and its connection with the properties of glasses such as ageing (e.g., decrease in the IS energy) or rejuvenation (e.g., increase in the IS energy). The challenge is twofold: firstly, glasses represent complex non-equilibrium states of matter that contain many-body interactions and strong disorder. This makes it difficult to develop parameter-free theories based on defects, because the definitions of defects in disordered materials are not unique and would require some phenomenological presumptions and fitting parameters^7^,^8^,^9^. Secondly, although computational modeling have yielded notable advances in our knowledge of complex materials at the atomistic scale, the traditional techniques (e.g., molecular dynamics (MD)) can only tackle problems at limited timescales and therefore face formidable challenges in probing materials behavior far from equilibrium.

The IS and the potential energy landscape (PEL) are known to be capable of providing a convenient framework to interpret complex phenomenology in disordered materials^10^,^11^,^12^,^13^, and more importantly, they allow focusing directly on energy variations, making it easier to compare with experiments (e.g., differential scanning calorimetry (DSC)) with minimal fitting parameters. In the PEL perspective, the elementary processes are the hopping between neighboring local minima. This consists of two stages; the activation stage (from the initial state to the connecting saddle state with the activation barrier *E*_A_), and the relaxation stage (from the saddle state to the final state with the energy relaxation *E*_R_), as illustrated in Fig. 1. Assuming at time *t* the IS energy of the system is *E*_IS_(*t*), one can then write down its evolution equation as:


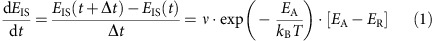


where 
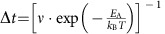
 represents the waiting time for hopping, and *ν* is the jump frequency which includes the entropic effect. Note that Fig. 1 is only a schematic illustration, and in disordered materials, each IS is connected to many different saddle states. Therefore all the possibilities need to be considered and equation (1) has to be solved in a mean-field sense as:


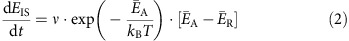


where the details of 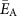 and 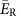 are discussed below. Given equation (2) one can probe how the IS of a glassy system evolves with time under different thermal protocols. In particular, whether the system ages or rejuvenates is determined by the sign of (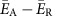): a negative sign corresponds to ageing (IS energy loss), while a positive sign represents rejuvenation (IS energy gain). In the following sections, we will identify the corresponding conditions where the system would undergo ageing, exhibit rejuvenation, or keep itself in the equilibrium state.

## Results

### Spectra of *E*_A_ and *E*_R_

To solve equation (2) we first have to know the dependence of the activation barrier (*E*_A_) and the energy of relaxation (*E*_R_) on *E*_IS_. Due to its disordered nature in glasses, both *E*_A_ and *E*_R_ are expected to display wide spectra, rather than few explicit values as in their crystalline counterparts. These spectra are obtained by the activation-relaxation technique (ART)^14^,^15^—a method of atomistic simulation known to be capable of capturing saddle point states and providing representative PEL samplings in amorphous systems, including high activation barrier events which are not accessible by MD simulations^16^,^17^. As an example here we consider a well-known glass former system Cu_56_Zr_44_ containing 2,000 atoms with a realistic many-body interacting potential^18^. The system is first equilibrated in a high temperature (2,000 K) liquid state and then cooled to 0 K with six different thermal conditions, including instant quench (IQ) and cooling with various rates ranging from 10^13^ K s^−1^ down to 10^9^ K s^−1^. Under such protocols six samples with the same composition but very different *E*_IS_ are obtained: slower cooling rates lead to lower IS energies. The samples are then input into ART to probe the *E*_A_ and *E*_R_ spectra.

**Figure 2:**
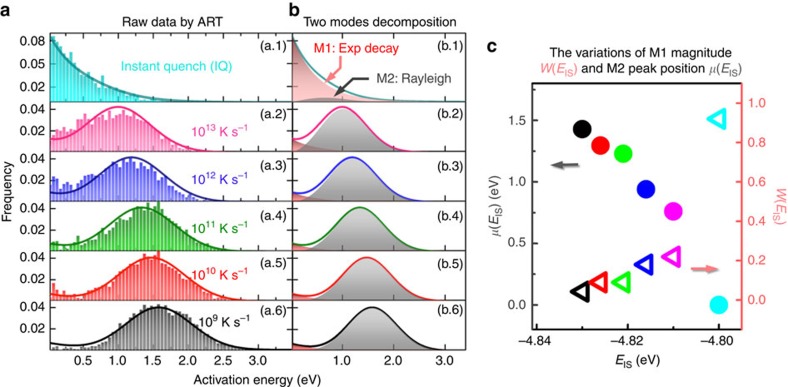
Dependence of activation energy spectra on cooling rates and EIS. (**a**) The raw histogram data on the activation barrier spectra in different samples obtained by ART. (**b**) The spectra can be decomposed into two modes, namely an exponential decay mode (M1), and a shifted-Rayleigh distribution (M2), whose expressions are displayed in **c**. The magnitude of M1, defined as *W*, and peak position of M2, defined as *μ*, are closely related to the system's IS energy. As IS energy decreases, *W*(*E*_IS_) becomes smaller, while *μ*(*E*_IS_) becomes larger.

Figure 2a shows the activation energy spectra obtained for these samples. Not surprisingly faster cooling rates yield smaller average *E*_A_, and similar behavior has also been observed in other different glassy systems^16^,^17^,^19^,^20^,^21^,^22^,^23^,^24^,^25^. More interestingly, it appears that the spectra can be decomposed into two different modes: an exponentially decaying mode at low energies (M1), and a shifted-Rayleigh distribution with a clear peak at higher energies (M2). Moreover, the decay rate of M1 and the width of M2 are nearly independent of the cooling rate. Given this the probability distribution function (PDF) of *E*_A_ can then be expressed as:


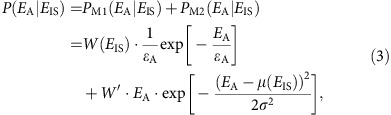


where *ɛ*_A_=0.5 eV, *σ*=0.49 eV, *W* (*E*_IS_) is the amplitude of M1, and *μ*(*E*_IS_) is the location parameter of M2. Note that *W*(*E*_IS_) and *μ*(*E*_IS_) are the only two independent variables to define the PDF explicitly in equation (3), because *W*′ (the relative weight of M2) can be calculated according to normalization condition of the PDF.

As seen in Fig. 2b, when the IS energy becomes lower (i.e., at a slower cooling rate), the amplitude of M1 becomes smaller and the peak of M2 shifts gradually to larger values. An interesting point we would like to note is that, although *W* (*E*_IS_) decreases very rapidly in the high cooling rate regime, it never disappears at low cooling rates. In the present study, M1 still persists with a fraction around 5% in the total weight even at the cooling rate of 10^9^ K s^−1^. Such persistence of M1 is probably a characteristic of ZrCu-based glassy system, since experiments^26^ also found extra low activation energy distributions in the same system. However in other systems, e.g., Pd_80_Si_20_ (ref. 26) and amorphous Si (ref. 17), there are no indications of the M1 mode.

In summary if the values of *W* (*E*_IS_) and *μ* (*E*_IS_) are provided, one can then determine the PDF in equation (3), which would further enable the calculation of the average barrier height in the thermally activated processes as:


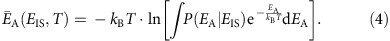


Due to the Boltzmann factor, the average activation barrier now becomes a function of both the IS energy and temperature. Note that equation (4) essentially represents the average of statistically independent events. Although it is an approximation, its validity and rationalization have been extensively discussed by Derlet *et al*.^27^,^28^.

As mentioned above, to solve equation(2) the spectrum of energy relaxation *E*_R_ also needs to be known. Interestingly, in contrast to *E*_A_, the spectrum of *E*_R_ was found to be quite insensitive to the IS energy (Supplementary Fig. 1), as was found in the simulation for amorphous Si (ref. 17). The significance of this point will be discussed below. We found that the spectra in all six samples show essentially the same exponential decay distribution,


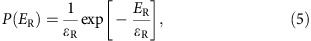


With 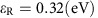. Therefore the average energy of relaxation can be calculated as:


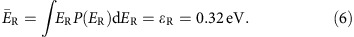


Note that there is no Boltzmann factor in equation (6), because the transition from the saddle state to the final state is only down-hill athermal relaxation^29^, not a thermally activated process.

### Ageing-rejuvenation crossover

In equation (2) whether the system undergoes ageing (d*E*_IS_/d*t*<0) or rejuvenation (d*E*_IS_/d*t*>0) depends on the sign of the difference between 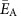 and 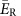 (defined as 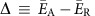). The ageing-rejuvenation crossover boundary is then governed by the balancing of activation and relaxation shown below in equation (7):


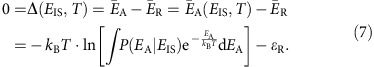


In other words, the solutions to equation (7) define a curve in *E*_IS_ *—T* space, on which the system is in equilibrium. As discussed above, if the forms of *W*(*E*_IS_) and *μ*(*E*_IS_) are provided, one can readily solve equation (7) and thus predict the equilibrium line. In the present study we have six different samples, the analyses of which show very clear and consistent trend, namely the decrease in *W*(*E*_IS_) and the increase in *μ*(*E*_IS_) as *E*_IS_ becomes smaller at lower cooling rates (seen in Fig. 2c). However we only have six data points, with inevitable statistical errors embedded in ART calculations, which are insufficient in determining the accurate forms of *W*(*E*_IS_) and *μ*(*E*_IS_). Ideally we should be able to gain more knowledge by preparing many more samples in a wider range of *E*_IS_, particularly those quenched at slower rates, and by repeating the analyses similarly done in Fig. 2. In principle, as stated by Kallel *et al*.^17^, a combination of ART method and Metropolis algorithm could relax the system to a deeper *E*_IS_ level. But it will take prohibiting amounts of computing time and therefore is impractical at this moment. Given the challenge, we formulated an ‘inverse' strategy in the present study. To be more specific, we first seek (via trial-and-error manner) appropriate functions of *W*(*E*_IS_) and *μ*(*E*_IS_) that can produce the correct equilibrium line through equation (7), and then compare the obtained functions with the limited data points in Fig. 2c to see whether they are consistent with the data or not.

**Figure 3:**
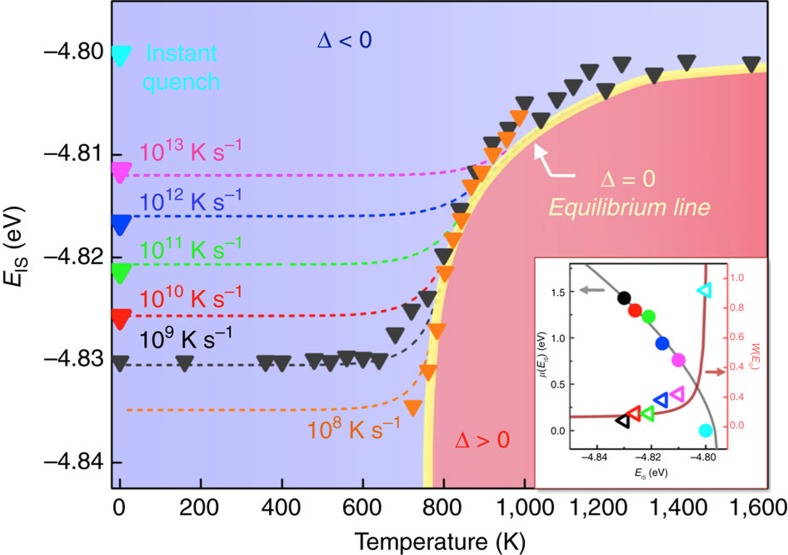
Regimes of ageing and rejuvenation in IS energy-temperature space. The triangle data points are the MD results at different thermal protocols. In order to keep the plot less busy, only the final quenched IS energy results are shown for the conditions of 10^10^ K s^−1^, 10^11^ K s^−1^,10^12^ K s^−1^,10^13^ K s^−1^, and instant quench (more MD data are shown in Supplementary Fig. 3). Yellow curve is the extracted equilibrium line from MD data. To reproduce the equilibrium line through equation (7), the expected *W* and *μ* should have the forms of 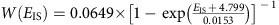, and 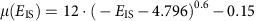, respectively, which are shown by the two curves in the inset plot. It can be seen that the previously obtained data points in Fig. 2c match very well with these functions. The dashed curves are the predicted cooling curves by equation (2) at different thermal protocols. The blue area corresponds to the ageing regime since δ is negative, while the red area represents the rejuvenation regime since δ is positive.

The equilibrium line in supercooled liquid can be extracted from MD results during the cooling studies^12^,^13^. Figure 3 shows the variations of IS energy at different temperatures in MD studies at cooling rates of 10^9^ K s^−1^ and 10^8^ K s^−1^. Given the intrinsic limitation on timescale in MD, in the present study we are only able to collect small amounts of data in a narrower temperature range for the condition of 10^8^ K s^−1^ (orange triangles in Fig. 3). At high temperatures, the system can easily get equilibrated and the IS energy is almost constant. However below 1,000 K, the IS energy becomes very sensitive to temperature and the required time for reaching equilibrium becomes exceedingly long as temperature decreases. When the timescale of cooling rate cannot keep up with intrinsic timescale in supercooled liquid, the system would jump out of equilibrium and eventually turn into the glassy state with a frozen IS (e.g., a flat array of black triangles at low temperatures in Fig. 3). From the MD data we can thus extract the equilibrium line, as shown by the yellow curve in Fig. 3.

As discussed above, in order to reproduce the equilibrium line through equation (7), we found the appropriate forms and shapes of *W*(*E*_IS_) and *μ*(*E*_IS_), which are shown by the two curves in the inset plot of Fig. 3 (more details in Supplementary Material Eqs 2,3). It can be seen that the previously obtained six sets of data points from independent ART studies are fully consistent with the assumed *W*(*E*_IS_) and *μ*(*E*_IS_). Such good agreements between two independent studies validate the current model and demonstrate that the nature of ageing-rejuvenation crossover in glassy system originates from activation (*E*_A_)—relaxation (*E*_R_) balancing in PEL. At lower temperature or higher IS energy, the average activation energy 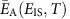 becomes smaller, leading to a negative Δ and the ageing regime (blue area), whereas at higher temperature or lower IS energy, Δ becomes positive, corresponding to the rejuvenation regime (red area).

### Origin of thermal hysteresis

To understand fully the dynamics of IS energy at arbitrary thermal protocols (including those non-equilibrium scenarios), one still needs to solve equation (2) under various conditions. As mentioned before, the entropic effect in equation (2) is included in the jump frequency as 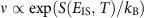. As first pointed out by Goldstein^30^ and Johari^31^ and later verified in many other studies^32^,^33^,^34^,^35^, the entropy of a glassy material is dependent on both *E*_IS_ and *T*. Although the quantitative relationship varies a bit in different studies, the general rule is that entropy becomes smaller at deeper IS energy or at lower temperature (more details in Supplementary Note 3). Having considered this, the numerical solutions to IS energy at different cooling rates can then be readily calculated (dashed curves in Fig. 3), which are found fully consistent with the independent MD simulation results at the same conditions.

Thermal hysteresis near the glass transition is a well-known phenomenon in amorphous materials^36^,^37^. DSC experiments show that, with the same rate of temperature change, heating process leads to a larger heat capacity peak at a higher temperature compared to the cooling process. Here we show that such a behavior can be naturally explained by our IS energy evolution model, i.e., equation (2).

**Figure 4:**
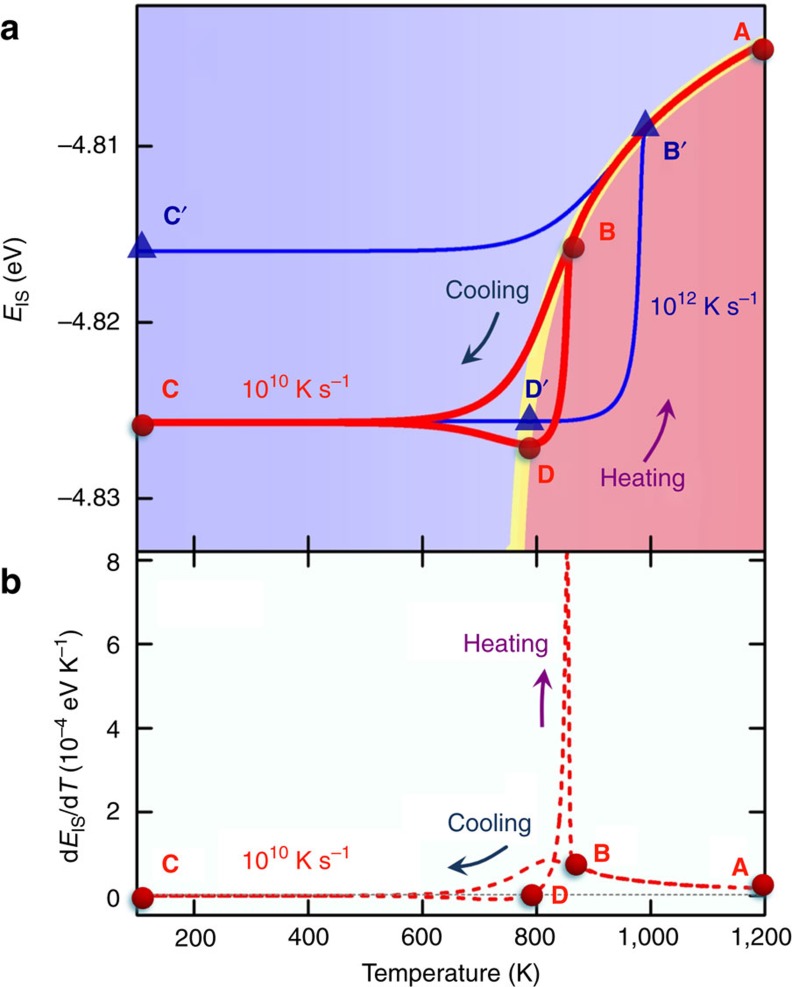
Origin of thermal hysteresis in cyclic thermal scanning. (**a**) *E*_*IS*_ evolutions at two different thermal protocols predicted by equation (2). Red curve represents a cooling (**A**→**B**→**C**) and heating (**C**→**D**→**B**→**A**) process with the 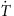 of 10^10^ K s^−1^. Blue curve represents a heating (**C**→**D′**→**B′**→**A**) and cooling (**A**→**B′**→**C′**) process with the 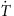 of 10^12^ K s^−1^. (**b**) The derivation of *E*_IS_ with respect to *T* associated with the first protocol shown in the upper panel. The heating process yields to a bigger peak at higher temperature than in the cooling, a known hysteresis phenomenon in glassy system.

The red curve in Fig. 4 (upper panel) shows a cooling (**A**→**B**→**C**) and heating (**C**→**D**→**B**→**A**) process at the rate of 10^10^ K s^−1^ predicted by equation (1). In a high temperature regime (e.g., **A**→**B**) the system can be equilibrated at a very short time scale and therefore the cooling curve coincides with the equilibrium line. At lower temperatures, however, the cooling rate of 10^10^ K s^−1^ is too fast to equilibrate the system, and the cooling curve deviates from the yellow line and enters the ageing regime (i.e., Δ<0 starting from point **B**. At this stage (**B**→**C**) *E*_IS_ is monotonically decreasing, and the reason why it looks almost flat below 500 K is that the jump frequency *ν* becomes smaller at a deeper IS energy or at a lower temperature, resulting in a very slow change in *E*_IS_. Now upon heating from point **c**, *E*_IS_ keeps monotonically decreasing until the curve hits the yellow line at point **D**, because at this stage (**C**→**D**) the system is still located in the Δ<0 regime (the reason it looks flat below 500 K is the same as described above). This in fact is the origin of the thermal hysteresis behavior.

Beyond the point **D** the system enters the Δ>0 regime and *E*_IS_ begins to increase. Due to the entropic effect the jump frequency *ν* becomes larger as both *E*_IS_ and temperature increase, therefore the increase in the IS energy becomes faster and faster until it hits again the equilibrium line at point **B**. It then follows the yellow line afterward. The dashed red curve in Fig. 4 (lower panel) is the derivative of *E*_IS_ with respect to *T* in this cooling-heating process. Clearly the key features of thermal hysteresis, namely a larger heat capacity peak at a higher temperature during heating than in cooling, are naturally reflected in this *E*_IS_ evolution model. It is also worth noting that because the process **B**→**C**→**D**→**B** is an off-equilibrium process, the accurate positions of those points are sensitive to the initial conditions and thermal protocols. For example Fig. 4 shows a heating (**C**→**D′**→**B′**→**A**) and cooling (**A**→**B′**→**C′**) process from the same point **C**, but at a different 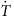 of 10^12^ K s^−1^. A higher heating rate makes the minimum at point **D′** shallower than at point **D**, and pushes the fast increase in *E*_IS_ to higher temperatures (from **D**→**B** to **D′**→**B′**). This also explains why higher heating rates lead to heat capacity peaks at higher temperatures in^36^. Beyond the point **B′**, even with the higher 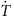 the system has enough time to reach equilibrium, and therefore **B′**→**A** coincides again with the yellow line. In the cooling process afterward, the higher cooling rate (10^12^ K s^−1^) makes the curve deviate from the equilibrium line earlier and end up with a higher *E*_IS_ at point **C′** than the initial point **C** prepared at a lower cooling rate of 10^10^ K s^−1^.

### Anomalous peaks in truncated thermal scanning

**Figure 5:**
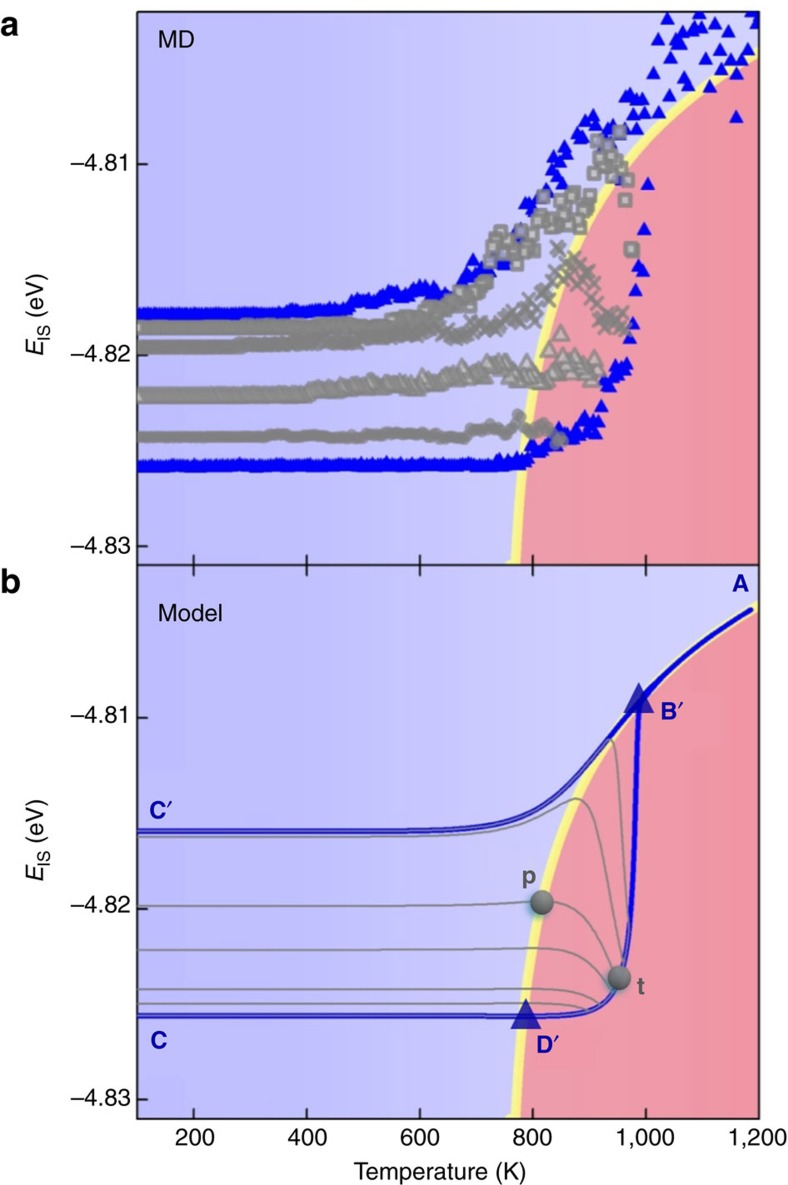
Predicted abnormal peaks in truncated thermal scanning and the validations by MD results. (**a**) MD simulations with 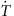 of 10^12^ K s^−1^, and with the bouncing back temperatures at 985, 970, 920 and 860 K, respectively (the grey data points from high to low). The MD data show clear peak structures precisely at the *E*_A_−*E*_R_ balancing line predicted by equation (7). (**b**) Model-predicted abnormal peak of *E*_IS_ at point **p** on the yellow line in the truncated thermal cycle (i.e., bouncing back temperature at **t** between **D′** and **B′**), which are well consistent with independent MD tests.

In the regular heating-cooling protocols, glasses are heated up to a well-equilibrated liquid state far beyond the glass transition temperatures and then cooled down back to low temperatures (e.g., **C**→**D′**→**B′**→**A**→**B′**→**C′** in Fig. 4). Under such protocols, *E*_IS_ shows a normal and monotonic decreasing behavior during the cooling process. However if the protocol is truncated, e.g., by terminating the heating process at an intermediate point between **D′** and **B′** and immediately starting the cooling process afterwards, our model would predict a non-monotonic variation of *E*_IS_ during cooling with a peak right on the activation-relaxation balancing line. The reason is again readily explained by equation (2). To be more specific let us consider a truncated protocol (at a fixed 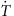 of 10^12^ K s^−1^) starting from the same point **C** as in Fig. 4 but with a bouncing back temperature at point **t**, as illustrated in the lower panel of Fig. 5.

Now when the cooling process just kicks off, because the system is still located in the Δ>0 regime, *E*_IS_ keeps increasing even if the temperature is decreasing. Once the curve hits and goes across the yellow line Δ turns into negative and *E*_IS_ starts to decrease, leaving behind a peak value of *E*_IS_ right at the yellow line (point **p**). Different bouncing back temperatures yield different curves, but they all display peaks at the activation-relaxation balancing line (lower panel in Fig. 5).

To verify such prediction, independent MD tests are employed at the same 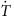 (10^12^ K s^−1^). As seen in the upper panel of Fig. 5, if the bouncing back temperature is very high (around 1,200 K), the MD displays a normal protocol behavior shown by the blue triangles. However when the heating processed are truncated at intermediate temperatures (between 800 K to 1,000 K), the MD data (grey points) clearly show peaks precisely located at yellow line predicted by equation (6). The good agreements here again demonstrate the underlying driving force of ageing or rejuvenation comes from the interplay between activation and relaxation on the PEL.

## Discussion

In the present study we develop a model to describe the dynamics of IS in glassy systems, which makes it possible to quantify the *E*_IS_ evolution in a self-consistent manner. It has been convincingly shown that system's ageing (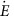
_IS_<0) or rejuvenation (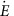
_IS_>0) is governed by the underlying competition between activation and relaxation on the PEL. As demonstrated in Fig. 3, the equilibrium line of the supercooled liquid is exactly the activation-relaxation balancing curve (i.e., Δ=0). We would like to note that, in obtaining the equilibrium line one only needs to know the *E*_A_ and *E*_R_ spectra, i.e., the distributions of *P*(*E*_A_*|E*_IS_) and *P*(*E*_R_*|E*_IS_). Because such distributions are simultaneously determined by the PEL and interactions between constituent particles in the materials, from this sense the model shown in the present work does not need to introduce pre-assumed mechanisms or further parameters, which could be an advantage for disordered materials modeling.

As shown in equation (5) and Supplementary Fig. 1, the distribution of the relaxation energy after passing through the saddle point is almost always the same, regardless of the initial state. The same feature was also observed in amorphous Si (ref. 17), and was suggested by internal friction experiments in a metallic glass as well^38^. In other words, by the time the system reaches the saddle point it forgets where it came from. Therefore, the correlations between elementary activations and relaxations in PEL, if any, are very weak, as evidenced by Supplementary Fig. 2 and in earlier studies by Swayamjyoti *et al*.^39^,^40^ and Kallel *et al*.^17^. The decoupling of activation and relaxation greatly simplifies the dynamics of IS migration through equation (2). Further implications of this critical feature will be discussed elsewhere. We would like to note that, the quantitative results in the present work rely upon two assumptions: (i) the independence of 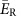 on 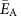 and *E*_IS_, and (ii) the accuracy of *P*(*E*_A_|*E*_IS_) over the entire range of *E*_IS_. Although the comparison with MD results in the cooling rate ranging from 10^13^s^−1^ to 10^8^s^−1^ suggest the two assumptions are valid, there is no guarantee that they still hold at lower levels of *E*_IS_ (i.e., at much lower cooling rates). However, we believe that the underlying framework of the present model, i.e., equation (2), is still valid, because equation (2) only describes the physics process of hopping in PEL and does not rely on the above mentioned two assumptions. Even if at deep levels of *E*_IS_ (e.g., at experimental quench rates) *P*(*E*_A_*|E*_IS_) were to be significantly different, and 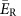 were to depend on *E*_IS_ and correlate with 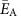, equation (2) would still provide a steady-state solution, although the detailed shape and position of such equilibrium line in *E*_IS_—*T* diagram could be different from what is shown now in Fig. 3 and given by equation (7).

The *E*_IS_ evolution model in the present study can also naturally explain the thermal hysteresis phenomenon observed in experiments. In addition, our model predicts an anomalous peak of *E*_IS_ in truncated thermal cycles, which has been verified by independent MD studies shown in Fig. 5. Such a success in prediction suggests that our model correctly captures the interplay between activation and relaxation on the PEL of the system, which are the underlying driving force in supercooled liquids and glasses. This in turn allows us to speculate about possible implications of such findings. For example during the stage **t**→**p** in Fig. 5, the IS energy keeps increasing even if the temperature is being reduced. This corresponds to negative heat capacity which might be captured in high-throughput DSC measurement. Interestingly experiments have found some non-monotonic behaviors in glassy systems under different thermal protocols, such as the crossover phenomena^41^,^42^ and the Kovac effect^43^. The most straightforward explanation for the crossover is that there should be more than one kinetic processes involved^41^,^42^. In other words a broad distribution of *E*_A_ is critical, and our present results provide fundamental support to such hypothesis.

It is also worth noting that in the present study we consider a metallic glass system Cu_56_Zr_44_ known as a fragile glass former, and its *E*_A_ spectra show two-mode decomposition. Due to the persistence of M1 mode, there exists a critical temperature (*T*_c_∼760 K) below which there is no solution to equation (7). In other words, below *T*_c_ equilibrium can never be reached, or equivalently ergodicity is broken. However recent studies^17^ on a strong glass former, amorphous silicon, showed that *E*_A_ spectra display only the M2 mode. Very interestingly, we find that if M1 is missing then there always is a solution to equation (7), or equivalently *T*_c_∼0 K. The asymptotic behavior as the system approaching *T*_c_ can also be explicitly obtained based on details of *P*(*E*_A_|*E*_IS_) and *P*(*E*_R_|*E*_IS_), as will be discussed later in a separate paper. It is also worth noting that, the well-known Vogel-Tammann-Fulcher (VFT) relationship (*η*=*A*exp[*B*/(*T*−*T*_0_)]) correlates the fragility of glass with a parameter *T*_0_, where *T*_0_ is a finite number for a fragile system and *T*_0_ approaches to 0 K for a strong system. The similarity between *T*_0_ and *T*_c_ thus builds very interesting and profound connections between the *E*_A_ spectra and the origin of fragility in glass formers. Admittedly there are new challenges remain to be addressed. For example, in the VFT model the *T*_0_ is attributed to the properties of the mega-basins in PEL^13^. But in the present work, with only neighboring basins considered, a similar role of *T*_c_ can also be obtained. Reconciling the two pictures, as well as identifying the structural correspondence to the different modes of M1 and M2, would warrant further studies.

## Methods

### Model set-up and art parameters

The studied Cu_56_Zr_44_ system contains 2,000 atoms, with an embedded-atom method (EAM) interaction^18^. The sizes of the system are 32.43 Å × 32.43 Å × 32.43 Å, with periodic boundary conditions employed to all three directions. In ART simulations, the initial perturbations are introduced to a small group of atoms with local connectivity^44^. Specifically, an atom is selected as the central atom, then random displacements are induced to this atom and its first nearest neighbors. The perturbation direction is randomly chosen, while the magnitude of displacement is fixed at 0.5 Å. The system is relaxed to the saddle point following the Lanczos algorithm^15^,^45^,^46^,^47^, when the curvature of PEL is less than −0.01 eV Å^−2^. The convergence to saddle point is reached when the force of the system is less than 0.05 eV Å^−1^. For each selected group of atoms, 10 ART searches with different random perturbation directions are employed. The 2,000 atoms system thus provides 20,000 searches in ART. Therefore six samples together contribute 120,000 ART searches. After removing the failed and redundant searches, on average around 2,500 different processes are identified for each sample.

### Data availability

The data that support the findings of this study are available from the corresponding author upon reasonable request.

## Additional information

**How to cite this article:** Fan, Y. *et al*. Energy landscape-driven non-equilibrium evolution of inherent structure in disordered material. *Nat. Commun.* **8,** 15417 doi: 10.1038/ncomms15417 (2017).

**Publisher's note:** Springer Nature remains neutral with regard to jurisdictional claims in published maps and institutional affiliations.

## Supplementary Material

Supplementary Information Supplementary Figures, Supplementary Notes and Supplementary References

## Figures and Tables

**Figure 1:**
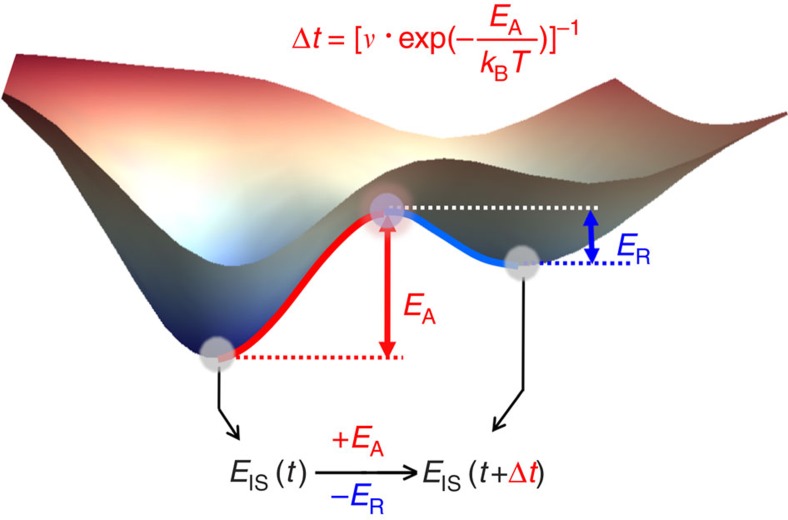
Schematic illustration of elementary hopping in potential energy landscape (PEL). The evolution of inherent structure (IS) energy is determined by the interplays between activation stage and relaxation stage in each elementary hopping. The *E*_IS_ change after hopping is given by *E*_A_−*E*_R_. Note that in disordered materials, each IS is connected to many different saddle states. Therefore in real calculation all the possibilities need to be considered, as discussed below in the text.
