# Supplementary material for: Energy landscape-driven non-equilibrium evolution of inherent structure in disordered material
Source: Nat Commun. 2017 May 19;8:15417. doi: 10.1038/ncomms15417 (PMC5454540; doi:10.1038/ncomms15417)
Supplement: Supplementary Information — Supplementary Figures, Supplementary Notes and Supplementary References [file ncomms15417-s1.pdf]

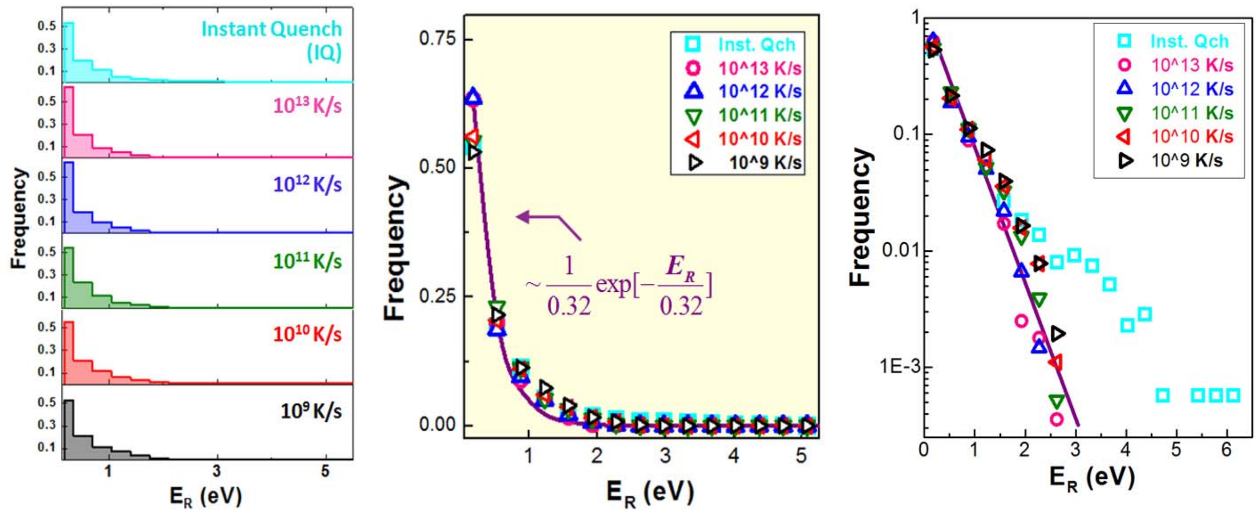

(a) (b) (c)

Supplementary Figure 1: The relaxation energy spectra E_R in six prepared sub-systems with different thermal protocols: (a) Histogram data; (b) The frequency distributions in regular plot; (c) The frequency distribution in the logarithmic scale plot. It can be seen that overall spectra are insensitive to the thermal histories and align well with an exponential decaying pattern with the decay constant of 0.32 *eV*. Note that instantly quenched sample show a long tail distribution below 1% level. The origin of such long tail distribution and its implication have been shown and discussed in an earlier study [1].

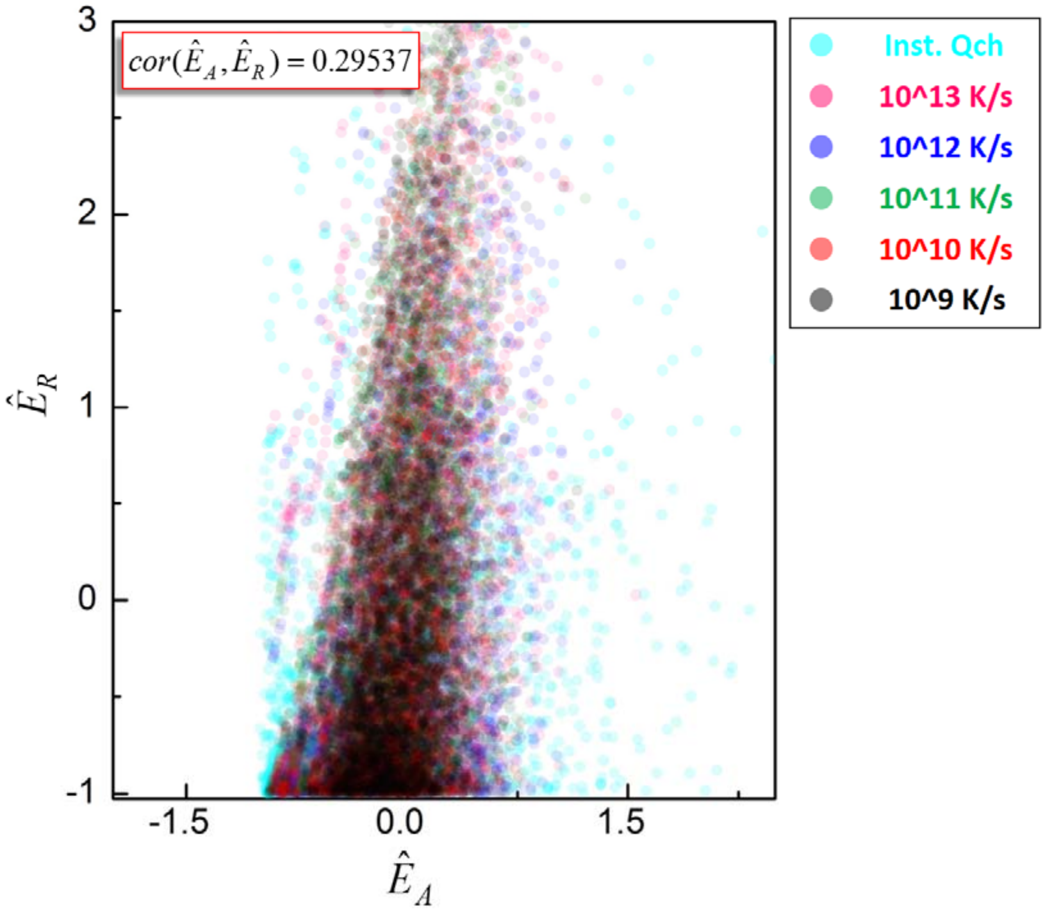

Supplementary Figure 2: The scattered plot between E_A and E_R in all the individual hopping events identified in the present study. To put all the data in the same plot, a simple unit reduction (see Supplementary Note 2 below) has been made. It can be seen that the correlations between activations and relaxations are weak.

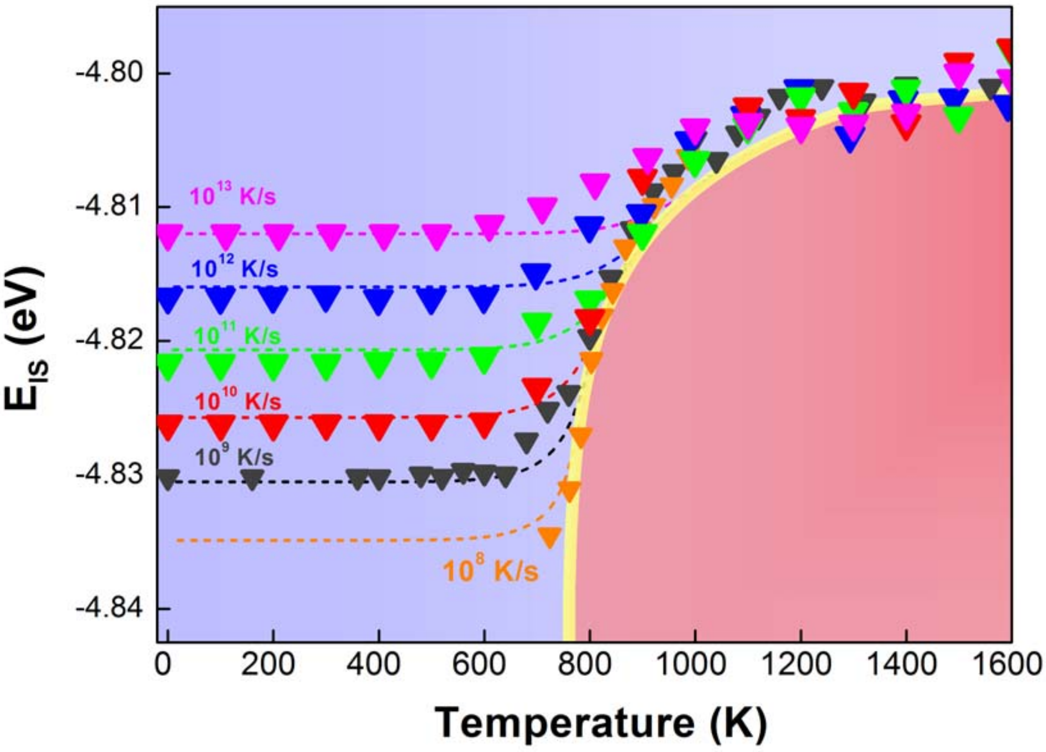

Supplementary Figure 3: More MD data on E_{IS} changes at different cooling rates. The data at higher cooling rates yield larger fluctuations due to the short simulation time, and at high temperatures the MD data show larger fluctuations as well. But overall the predictions by Eq. (2) and MD studies align well with each other

Supplementary Material Note 1: Dependence of E_R on E_{IS}

The raw data provided by ART on the energy relaxation E_R from saddle states to final states are shown in Supplementary Figure. 1.

In stark contrast to the activation energy spectra E_A, the distribution of the relaxation energy E_R from saddle states to final states appears to be insensitive to E_{IS} and thermal histories. The E_R spectra in all six samples show a similar exponential decaying pattern with a decay constant around 0.32 *eV*. Recent studies show that various thermal protocols could lead to different tail structures in the distributions [1], which is believed to have correlations with the intermittent patterns in X-ray photon correlation spectroscopy (XPCS) experiments [2]. But such difference can only be observed in a log-scale [1] and as clearly seen in Fig. S1.c, the overall distributions in different thermal systems all collapse with each other and they all have the same average value of 0.32 *eV*. This must be because the process of reaching the saddle point is highly plastic, with some atoms getting displaced by as much as 1 Å or more, way beyond the elastic limit. In fact the independence of E_R on E_{IS} has also been observed in a different glassy system [3]. Therefore the processes of activation and relaxation are essentially decoupled. This is an important result which greatly simplifies the dynamics of IS migration.

To better see the relationship between E_A and E_R, we made a scatters plot between the two variables shown in Supplementary Figure. 2. In order to put all the data points into the same plot, we made very simple unit reductions:

$$\hat{E}_A \equiv \frac{E_A - < E_A >}{< E_A >}, \quad \hat{E}_R \equiv \frac{E_R - < E_R >}{< E_R >} \qquad \text{(Supplementary Equation. 1)}$$

Apparently such reduction would not change the nature of correlation between E_A and E_R. Please note that in Supplementary Figure. 2, as discussed in the manuscript, $\hat{E}_A$ and $\hat{E}_R$ are not

uniform random variables. In contrast they follow either exponential decaying distributions or the combination of exponential and shifted-Rayleigh distributions. Therefore the distributions of the data points in the plot are not uniform: (i) when we see from the y-axis the most data points are near the bottom, because $\hat{E}_R$ follows exponential decaying distribution; and, (ii) when see from the x-axis most data points are near $\hat{E}_A \sim 0$, because as seen in Fig.2.a in the main text, except for the instantly quenched sample, all other 5 samples show well-defined peaks in the E_A spectra.

It can be seen that, *the majority* of the scattered data points (the thick area in the plot) approximately yields a symmetric shape with respect to the $\hat{E}_A = 0$ line. In other words, for any given value of $\hat{E}_R$, the corresponding average value of $\hat{E}_A$ along that horizontal line would yield the same number. Or alternatively, the average value of $\hat{E}_A$ does not depend on the value of $\hat{E}_R$, i.e. the two variables do not correlate with each other. Admittedly, we do find that when $\hat{E}_R$ becomes very large, based on the data, the two variables seem to be slightly coupled to each other (the data points are slightly slanted to the right), although the Pearson coefficient value of 0.295 suggests the correlation is very weak. We believe there are two possible reasons: (i) As discussed above, $\hat{E}_R$ follows exponential decaying distribution, therefore there are much less data points in the large $\hat{E}_R$ regime, and consequently we don't have sufficient statistics in that regime yet; (ii) $\hat{E}_A$ and $\hat{E}_R$ are weakly correlated in the large $\hat{E}_R$ range. In fact, we would also like to stress that, in Kallel *et al*'s study on amorphous Si [3], the correlations between the E_A and E_R distributions have been examined and found to be independent. In other words, "*the height of a barrier does not provide any information regarding the energy of the final minimum*" [3]. Therefore, such two features, namely (1) the independence of E_R on E_{IS}, and (2) the

independence (or at least weak correlation) between E_R and E_A, could be universal in amorphous systems.

Supplementary Material Note 2: Form of the E_A spectra

In order to reproduce the equilibrium line by solving Eq. (7) we found that the forms of the E_A spectra,

$$W(E_{IS}) = 0.0649 \times \left[1 - \exp(\frac{E_{IS} + 4.799}{0.0153})\right]^{-1} \qquad \text{(Supplementary Equation. 2)}$$

$$\mu(E_{IS}) = 12 \cdot (-E_{IS} - 4.796)^{0.6} - 0.15 \qquad \text{(Supplementary Equation. 3)}$$

are appropriate. They agree well with the MD data as shown in the inset of Fig. 3.

Supplementary Material Note 3: Jump frequency *v* and entropy $S(E_{IS},T)$

As discussed in the main text, the entropic effect in Eq. (1) is included in the jump frequency as $\nu \propto \exp(S(E_{IS},T)/k_B)$. Goldstein [4] and Johari [5] pointed out that the entropy of glassy material is dependent on both E_{IS} and T, and it has been supported by many other studies [6, 7, 8, 9]. In the PEL perspective, the entropy can be divided into two parts [10]: the first part comes from the oscillations inside a local minimum state, namely the vibrational entropy; while the second part is related to the number of distinct local minimum states (*i.e.* degeneracy) at a certain energy level, also known as the configurational entropy.

The configurational entropy is very sensitive to E_{IS} and widely believed to vary in a parabola shape as $S_{conf}(E_{IS}) = \alpha_c - [\beta_c \cdot (E_{IS} - E_0)]^2$ [7, 11, 12]. The temperature dependence of entropy mainly stems from the vibrational contribution S_{vib}. In principle by diagonalizing the

system's Hessian matrix at a local minimum state and getting the eigen-frequencies one would be able to calculate the vibrational entropy. However there is not a well-accepted expression developed for the dependence of S_{vib} on temperature, due to the complex density of states in glasses [13] and anharmonicity [11]. Given such challenge, as well as the general rule that vibrational entropy becomes larger as temperature increasing, in the present study we just assume S_{vib} varies in a linear manner as $S_{vib}(T) = \alpha_v + \beta_v (T - T_0)$ for the simplicity of calculation. The jump frequency can then be expressed as:

$$\begin{aligned} \nu &\propto \exp(S(E_{IS}, T)/k_B) \propto \exp[S_{vib}(T) + S_{conf}(E_{IS})] \\ &= \nu_0 \cdot \exp[\beta_v (T - T_0)] \cdot \exp\left\{-[\beta_c \cdot (E_{IS} - E_0)]^2\right\} \end{aligned} \quad \text{(Supplementary Equation. 4)}$$

In the present study, the parameters are set as $\nu_0 = 9.4 \times 10^9\, s^{-1}, \beta_v = 0.02\ K^{-1}, T_0 = 300\ K$, $\beta_c = 100\ eV^{-1}, E_0 = -4.8073\ eV$.

We would like to stress that the key result of the present work (*i.e.* the yellow line in Fig. 3 in the main text) has nothing to do with the parameters employed in Supplementary Equation. 4. The equilibrium line in Fig. 3 is predicted by Eq. (7) in the main text, which only relies on E_A and E_R spectra directly provided by atomistic sampling on PEL and does not need any phenomenological presumptions or fitting parameters. In contrast, the above-mentioned parameters in Supplementary Equation. 4 only determine the quantitative rate on how the system is deviating from equilibrium. The critical qualitative features (*e.g.* thermal hysteresis in Fig. 4 and abnormal peaks in truncated thermal cycles in Fig. 5), however, will not be affected because all these behaviors originate from the aging-rejuvenation crossover, which is solely governed by the yellow line in Fig. 3.

We would also like to point out that, for the parameters used now in Supplementary Equation. 4 and particularly the formalism of S_{vib}, there is still plenty of room to further adjust and optimize them. For example in the present study, for the simplicity of calculation, we separate the temperature dependence and E_{IS} dependence as $S_{vib}(T)$, and $S_{conf}(E_{IS})$, respectively. In reality, however, situation could be much more complicated. For example, Rodney *et al*'s studies [9] suggest that the vibrational entropy is also largely influenced by E_{IS}. This would yield a coupling effect between T and E_{IS} on the entropy, which makes the calculation more difficult. But again as discussed above, the formalism of entropy and its parameters will not change the qualitative features. We regard that as numerical optimizations and will not further discuss that in the context of present work.

Supplementary References:

1. Fan Y, Iwashita T, Egami T. Crossover from Localized to Cascade Relaxations in Metallic Glasses. *Physical Review Letters* **115**, 045501 (2015).
2. Evenson Z*, et al.* X-Ray Photon Correlation Spectroscopy Reveals Intermittent Aging Dynamics in a Metallic Glass. *Physical Review Letters* **115**, 175701 (2015).
3. Kallel H, Mousseau N, Schiettekatte F. Evolution of the Potential-Energy Surface of Amorphous Silicon. *Physical Review Letters* **105**, 045503 (2010).
4. Goldstein M. Viscous liquids and the glass transition. V. Sources of the excess specific heat of the liquid. *The Journal of Chemical Physics* **64**, 4767-4774 (1976).
5. Johari GP. On the heat capacity, entropy and 'glass transition' of vitreous ice. *Philosophical Magazine* **35**, 1077-1090 (1977).
6. Yan Q, Jain TS, de Pablo JJ. Density-of-States Monte Carlo Simulation of a Binary Glass. *Physical Review Letters* **92**, 235701 (2004).
7. Sastry S. The relationship between fragility, configurational entropy and the potential energy landscape of glass-forming liquids. *Nature* **409**, 164-167 (2001).
8. Johari GP. A resolution for the enigma of a liquid's configurational entropy-molecular kinetics relation. *The Journal of Chemical Physics* **112**, 8958-8969 (2000).

9. Koziatek P, Barrat J-L, Derlet P, Rodney D. Inverse Meyer-Neldel behavior for activated processes in model glasses. *Physical Review B* **87**, 224105 (2013).

10. Berthier L, Biroli G. Theoretical perspective on the glass transition and amorphous materials. *Reviews of Modern Physics* **83**, 587-645 (2011).

11. Büchner S, Heuer A. Potential energy landscape of a model glass former: Thermodynamics, anharmonicities, and finite size effects. *Physical Review E* **60**, 6507-6518 (1999).

12. Debenedetti PG, Stillinger FH. Supercooled liquids and the glass transition. *Nature* **410**, 259-267 (2001).

13. Phillips WA, Buchenau U, Nücker N, Dianoux AJ, Petry W. Dynamics of glassy and liquid selenium. *Physical Review Letters* **63**, 2381-2384 (1989).
